# Evaluating the Need to Address Digital Literacy Among Hospitalized Patients: Cross-Sectional Observational Study

**DOI:** 10.2196/17519

**Published:** 2020-06-04

**Authors:** Hanna Vollbrecht, Vineet Arora, Sebastian Otero, Kyle Carey, David Meltzer, Valerie G Press

**Affiliations:** 1 Pritzker School of Medicine University of Chicago Chicago, IL United States; 2 Section of General Internal Medicine Department of Medicine University of Chicago Chicago, IL United States; 3 Section of Hospital Medicine Department of Medicine University of Chicago Chicago, IL United States

**Keywords:** health literacy, digital literacy, hospitalization, technology

## Abstract

**Background:**

Technology is a potentially powerful tool to assist patients with transitions of care during and after hospitalization. Patients with low health literacy who are predisposed to poor health outcomes are particularly poised to benefit from such interventions. However, this population may lack the ability to effectively engage with technology. Although prior research studied the role of health literacy in technology access/use among outpatients, hospitalized patient populations have not been investigated in this context. Further, with the rapid uptake of technology, access may no longer be pertinent, and differences in technological capabilities may drive the current digital divide. Thus, characterizing the digital literacy of hospitalized patients across health literacy levels is paramount.

**Objective:**

We sought to determine the relationship between health literacy level and technological access, use, and capability among hospitalized patients.

**Methods:**

Adult inpatients completed a technology survey that asked about technology access/use and online capabilities as part of an ongoing quality of care study. Participants’ health literacy level was assessed utilizing the 3-question Brief Health Literacy Screen. Descriptive statistics, bivariate chi-squared analyses, and multivariate logistic regression analyses (adjusting for age, race, gender, and education level) were performed. Using Bonferroni correction for the 18 tests, the threshold *P* value for significance was <.003.

**Results:**

Among 502 enrolled participants, the mean age was 51 years, 71.3% (358/502) were African American, half (265/502, 52.8%) were female, and half (253/502, 50.4%) had at least some college education. Over one-third (191/502, 38.0%) of participants had low health literacy. The majority of participants owned devices (owned a smartphone: 116/173, 67.1% low health literacy versus 235/300, 78.3% adequate health literacy, *P*=.007) and had used the Internet previously (143/189, 75.7% low health literacy versus 281/309, 90.9% adequate health literacy, *P*<.001). Participants with low health literacy were more likely to report needing help performing online tasks (133/189, 70.4% low health literacy versus 135/303, 44.6% adequate health literacy, *P*<.001). In the multivariate analysis, when adjusting for age, race, gender, and education level, we found that low health literacy was not significantly associated with a lower likelihood of owning smartphones (OR: 0.8, 95% CI 0.5-1.4; *P*=.52) or using the internet ever (OR: 0.5, 95% CI 0.2-0.9; *P*=.02). However, low health literacy remained significantly associated with a higher likelihood of needing help performing any online task (OR: 2.2, 95% CI 1.3-3.6; *P*=.002).

**Conclusions:**

The majority of participants with low health literacy had access to technological devices and had used the internet previously, but they were unable to perform online tasks without assistance. The barriers patients face in using online health information and other health information technology may be more related to online capabilities rather than to technology access. When designing and implementing technological tools for hospitalized patients, it is important to ensure that patients across digital literacy levels can both understand and use them.

## Introduction

Technology-based interventions have the potential to improve care transitions; however, they can also exacerbate existing health disparities. For instance, hospitalization represents a time of vulnerable care transitions [1] when technology-based interventions could improve patient engagement and outcomes. Examples include patient portals [2], educational videos [3,4], mobile apps [5], telehealth [6], and remote monitoring [7], which have the possibility of improving patient-provider communication and patient education at the time of hospitalization and discharge, when patients may be coping with a new diagnosis or needing assistance with controlling a chronic disease. However, these interventions can only be broadly effective if all patients are able to access, use and effectively understand them. Historically, the digital divide concept was characterized by differences in access to technology and largely driven by socioeconomic status (SES), age, and race [8]. Currently, with increasing access to technology-based devices, a shift to a digital capability divide may be even more salient [9].

Prior research has shown that health literacy is an important contributor to the digital capability divide, with patients with lower health literacy being 7%-47% less likely to access and have the ability to use technology [10,11]. At the same time, patients with low health literacy have worse health outcomes [12], increased risk of poor vision [13], longer hospital stays [14], increased hospital-to-home transitional care needs [15], and increased readmission risk, especially among older patients [16]. Therefore, technology-based interventions could be a mechanism to improve long-term outcomes for hospitalized patients with low health literacy, while adding complexity for some patients. Research investigating the relationship between health literacy and technology has been primarily conducted among community-dwelling and outpatient populations but has not been evaluated among hospitalized patients.

The relationship between health literacy and technology use may be particularly relevant for hospitalized patients, since many care transition interventions rely on technology. Prior research has also suggested that health literacy is dynamic, with hospitalization representing a period when health literacy may acutely decrease [17]. It is also very likely that technology access and capabilities are not static, but dynamic. Hospitalization provides an important assessment time point to understand if and how technology can be utilized to improve health. Finally, hospitalized patients likely differ from those in community-dwelling or outpatient settings, making them an important population to characterize. Therefore, we sought to determine the relationship between health literacy level and technology access, use, and digital capabilities among hospitalized adult general medicine patients.

## Methods

We performed a cross-sectional observational study among adult inpatients at the University of Chicago Medicine as part of a large, ongoing study of inpatient quality of care [18]. Inclusion criteria included being hospitalized on a general medicine service, 18 years or older, and English-speaking. Exclusion criteria included an inability to provide consent and prior participation in the study. The University of Chicago Biological Sciences Division Institutional Review Board approved this protocol (#IRB16-0763).

We used a survey that was comprised primarily of national benchmarked Pew Research Center survey questions [19] that were categorized into 3 domains of technology: access, use, and capabilities. All variables in these domains were binary (yes/no). To assess technology access, participants were asked whether they owned a smartphone, computer, or tablet; whether they had a texting plan; and whether they had Wi-Fi at home. To assess technology use, participants were asked if they used the internet and whether they used the internet for health-related reasons. To assess digital capabilities, participants were asked if they knew how to perform a given online task or if they would need help. A summary measure was constructed for needing help with any online task, in which participants were categorized as needing help if they responded yes to needing help with one or more tasks. Participants were assigned as having either low or adequate health literacy based on the 3-question Brief Health Literacy Screen (BHLS). Each item in the BHLS is scored on a Likert scale from 0 to 4, with a score of 2 or less on any item identifying a participant as having low health literacy [20]. The BHLS and technology survey were orally administered.

Differences in technology access, use, and capabilities by health literacy level were analyzed using chi-squared tests. Multivariate logistic regression analyses were conducted to determine differences in technology access, use, and capabilities adjusting for health literacy (binary), age (continuous), gender (binary), race (white, non-Hispanic black, other), and education (less than any college versus some college or more). All analyses were performed using STATA version 15.0 (StataCorp), with *P*<.003 defining statistical significance based on Bonferroni correction.

## Results

**Figure 1:**
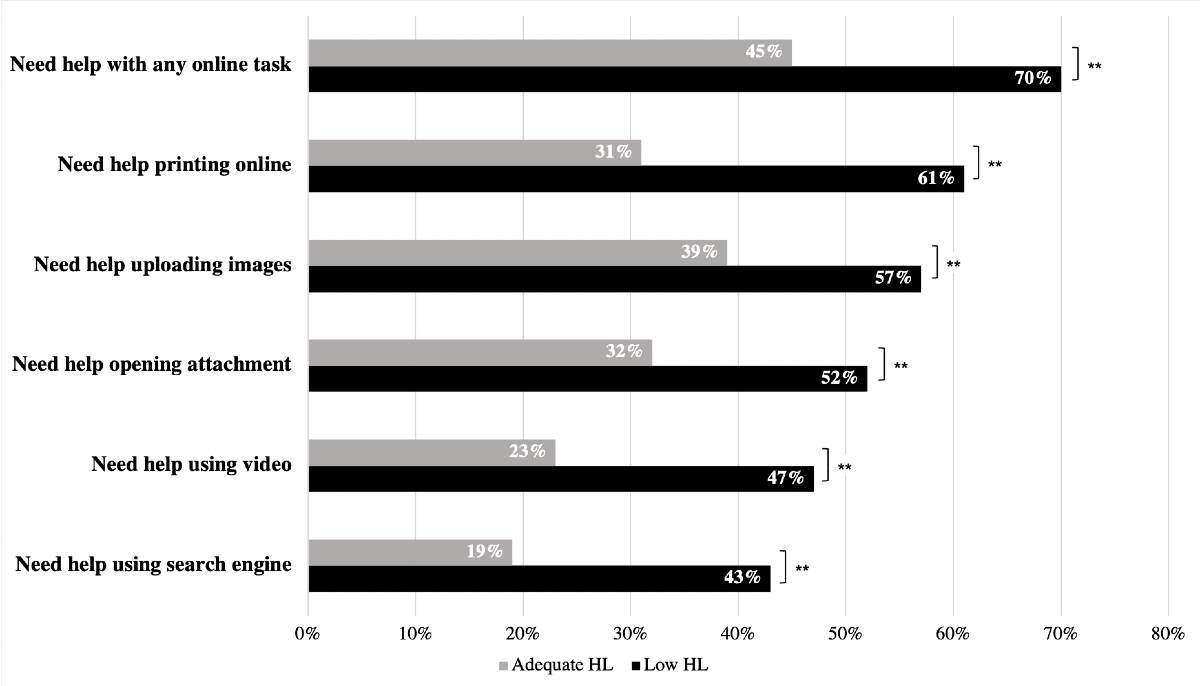
Percent of participants that report needing help with online tasks by health literacy (HL) level. ** demonstrates significance of *P*<.003 for Bonferroni correction. Raw numbers of participants needing help with each task are as follows: Need help with any online task – adequate HL 135/303 (44.6%), low HL 133/189 (70.4%); Need help printing online – adequate HL 95/303 (31.4%), low HL 116/189 (61.4%); Need help uploading images – adequate HL 119/303 (39.3%), low HL 107/189 (56.6%); Need help opening attachment – adequate HL 96/303 (31.7%), low HL 99/189 (52.4%); Need help using video – adequate HL 61/257 (23.7%), low HL 81/172 (47.1%); Need help using search engine – adequate HL 59/303 (19.5%), low HL 82/189 (43.4%).

From January 30, 2014 through May 10, 2018, 502 participants were enrolled in the study and completed the survey. Of these 502 participants, the mean age was 51 years, 358 (71.3%) were Black (non-Hispanic), 265 (52.8%) were female, and 253 (50.4%) had at least some college education. Out of 502 participants, 191 (38.0%) had low health literacy. Compared to participants with adequate health literacy, participants with low health literacy were less likely to own a desktop (49/191 [25.7%] low health literacy versus 130/311 [41.8%] adequate health literacy, *P*<.001) or laptop (63/191 [33.0%] versus 173/311 [55.6%], *P*<.001). In contrast, there was no significant difference in ownership of tablets (64/191 [33.5%] versus 139/311 [44.7%], *P*=.01) or smartphones (116/173 [67.0%] versus 235/300 [78.3%], *P*=.007) by health literacy level. Participants with low health literacy were less likely to report using the internet ever (143/189 [75.7%] versus 281/309 [90.9%], *P*<.001), daily internet use (91/189 [48.1%] versus 210/309 [68.0%], *P*<.001), or searching for health information online (95/155 [61.3%] versus 222/288 [77.1%], *P*<.001). Participants with low health literacy were more likely to report needing help for all online tasks queried (Figure 1).

In multivariate analysis, we found that health literacy remained significantly associated with some aspects of technology access/use, but not others (Table 1). For example, low health literacy was still associated with a lower likelihood of owning a laptop (*P*<.001), but not with owning a smartphone (*P*=.50). Low health literacy level was not significantly associated with a lower likelihood of internet use (*P*=.02) or searching for health information online (*P*=.10). Low health literacy was associated with a higher likelihood of needing help with online tasks overall. Further results from multivariate logistic regression analyses are provided in Multimedia Appendices 1 and 2.

**Table 1:** The relationship of low health literacy and technology access, use, and capabilities^a^.

Low health literacy^b^		OR^c^ (95% CI)	*P* value	AOR^d^ (95% CI)	*P* value
**Technology access**					
	Own desktop	0.5 (0.3-0.7)	<.001	0.6 (0.4-0.98)	.04
	Own laptop	0.4 (0.3-0.6)	<.001	0.5 (0.3-0.8)	.001
	Own tablet	0.6 (0.4-0.9)	.01	0.8 (0.5-1.2)	.33
	Own smartphone	0.6 (0.4-0.9)	.01	0.8 (0.5-1.4)	.52
	Wi-Fi at home	0.4 (0.3-0.7)	<.001	0.7 (0.4-1.0)	.07
	Text messaging plan-any	0.3 (0.2-0.6)	<.001	0.3 (0.2-0.7)	.002
	Unlimited text plan	0.5 (0.3-0.8)	.007	0.6 (0.3-1.0)	.05
**Technology use**					
	Ever internet use	0.3 (0.2-0.5)	<.001	0.5 (0.2-0.9)	.02
	Daily internet use	0.4 (0.3-0.6)	<.001	0.6 (0.4-0.97)	.04
	Search health info online	0.5 (0.3-0.7)	<.001	0.7 (0.4-1.1)	.11
	Post health info online	0.6 (0.3-0.997)	.05	0.6 (0.4-1.0)	.07
	Download app	0.6 (0.3-0.9)	.02	0.8 (0.5-1.4)	.40
**Technology capabilities**					
	Need help with any online task^e^	3.0 (2-4.3)	<.001	2.2 (1.3-3.6)	.002
	Need help to print online materials	3.5 (2.4-5.1)	<.001	2.7 (1.7-4.4)	<.001
	Need help to upload images	2.0 (1.4-2.9)	<.001	1.4 (0.9-2.2)	.20
	Need help to open attachment	2.4 (1.6-3.4)	<.001	1.7 (1.1-2.8)	.03
	Need help to use video	2.9 (1.9-4.3)	<.001	2.5 (1.4-4.2)	.001
	Need help to use search engine	3.2 (2.1-4.7)	<.001	2.1 (1.3-3.5)	.003

^a^Using Bonferroni correction for the 18 tests, the *P* value threshold for significance is <.003.

^b^Low health literacy is a binary variable where low HL = 0 denotes participants with adequate health literacy and low HL = 1 denotes participants with low health literacy.

^c^OR: unadjusted odds ratio.

^d^AOR: best fit adjusted odds ratio (adjusted for age [continuous], gender [binary], race [white, black, other], and education [less than any college versus some college or more]).

^e^Compilation of all items beginning with “Need help…”, with participants categorized as needing help if they responded Yes to needing help with 1 or more of the online tasks listed.

## Discussion

We found that despite most participants having access to smartphones and using the internet, hospitalized patients with low health literacy were significantly more likely to need help with online tasks. This raises the question of whether inpatients with low health literacy are able to effectively utilize technology, even if they have access to do so. Although our study suggests that certain devices, such as smartphones, may have appealing interfaces for patients across literacy levels, it also underlines that access is not synonymous with ability. This is consistent with a recent study showing disparities in hospitalized patients’ interest in patient portals [21]. Patients who were older, African-American, non-English speaking, or homeless were less likely to want to use patient portals and the second most commonly cited reason for this was an inability to use the internet. If technology is to be implemented more broadly during and after hospitalization for patient use, we must do so in a way that is palatable, engaging, feasible, and equitable for diverse populations. It is not enough to design these resources in a health-literate manner; we must also ensure that patients have the necessary digital skills to utilize them. Interestingly, results from multivariate analyses suggested that in the cases where health literacy is no longer associated with certain technological capabilities (specifically, using a search engine and uploading images), age and education are significantly associated. This suggests that in years to come, as more people are raised with technology, the digital divide will likely shrink. Examining trends of digital capabilities over the period of our study would be interesting future work.

Some hospitals are measuring health literacy among their patients to identify those who may be at high risk for nonunderstanding and poorer health outcomes [22]. In addition, our findings suggest that it may be important to assess digital literacy if hospitals are promoting the use of technology for patients’ self-care. It is also possible that a universal approach to digital literacy could be useful. Health literacy universal precautions have been proposed; they encourage providers and health care systems to approach all patients with the assumption that they may not understand health information. This is based on the idea that health literacy is a dynamic process that is not only determined by individuals’ abilities but also the complexities of the system [17]. In the same manner, hospitals could approach all patients with universal digital literacy precautions, ensuring that interventions are designed to be accessible and usable for patients across literacy levels and that patients have the ability to utilize technology as part of implementation. Although this requires up-front increased personnel to educate patients, long-term, technological resources could be cost-saving [23].

Recently, electronic health (eHealth) literacy has gained traction as an important aspect of patients’ ability to obtain and use information from online sources. eHealth literacy represents a complex interplay between multiple literacies, including health literacy and digital literacy [24], which we measured in this study. However, these two literacies alone likely do not adequately encompass all the skills necessary to effectively utilize technology-based health resources. Future studies could use eHealth literacy assessment tools to determine if and when eHealth literacy should be assessed prior to prescribing technology-based interventions in the hospital setting. Additionally, a recent study found that health literacy and eHealth literacy were not significantly correlated [25], suggesting that both may need to be studied to determine patients’ abilities to use technology-based health resources. Future studies could aim to examine eHealth literacy among hospitalized patients and whether health literacy, digital literacy, and eHealth literacy are correlated.

Limitations of our study include being a single-site study and using self-reported measures of technology access, use, capabilities, and health literacy. Further, our study did not measure eHealth literacy directly. Future studies could focus specifically on the eHealth literacy of hospitalized patients and how it relates to their ability to use health technologies during and after hospitalization.

In summary, our results indicated that health literacy is an important contributor to the digital capability divide among hospitalized patients and helped to identify areas of future research. Technology provides both opportunities for improvement during care transitions and potential pitfalls. To mitigate these pitfalls, health literacy and digital literacy should be accounted for when considering how best to implement technology-based interventions across hospital-to-home transitions of care.

## Appendix

Multimedia Appendix 1 Results of multivariate logistic regression analyses in which low health literacy (HL) adjusted odds ratio (AOR) was significant.

Multimedia Appendix 2 Results of multivariate logistic regression analyses in which low health literacy (HL) adjusted odds ratio (AOR) was not significant.

## Abbreviations

BHLS: Brief Health Literacy Screen
eHealth: electronic health
HL: health literacy
SES: socioeconomic status
