# Supplementary material for: Evaluating the Need to Address Digital Literacy Among Hospitalized Patients: Cross-Sectional Observational Study
Source: J Med Internet Res. 2020 Jun 4;22(6):e17519. doi: 10.2196/17519 (PMC7303835; doi:10.2196/17519)
Supplement: Multimedia Appendix 1 [file jmir_v22i6e17519_app1.docx]

**Multimedia Appendix 1**. Results of multivariate logistic regression analyses in which low health literacy (HL) adjusted odds ratio (AOR) was significant.

| **Dependent variable^a^** | **Low HL AOR^b^ (95%CI)**  ***P*-value** | **Control variables OR^c^ (95%CI),**  ***P*-value** | **Constant**  **(95%CI)**  ***P*-value** | **Df** | **Chi^2^**  **(*P*-value)** | **Log likelihood** |
| --- | --- | --- | --- | --- | --- | --- |
| Own laptop | 0.49  (0.32, 0.75)  *P*=.001 | -Age: 0.98 (0.97, 1.0), *P*=.007 | 3.43  (1.49, 7.91)  *P*=.004 | 6 | 78.2 (*P*<.001) | -288.0 |
|  |  | -Gender: 1.2 (0.79, 1.8), *P*=.42 |  |  |  |  |
|  |  | -Race  Black: 0.35 (.21, .58), *P*<.001  Other: 0.33 (0.11, 1.0), *P*=.05 |  |  |  |  |
|  |  | -Education: 2.66 (1.8, 4), *P*<.001 |  |  |  |  |
| Text messaging plan-any | 0.34  (0.17, 0.67)  *P*=.002 | -Age: 0.93 (0.91, 0.95), *P*<.001 | 993  (146, 6570)  *P*<.001 | 6 | 64.3  (*P*<.001) | -123.6 |
|  |  | -Gender: 1.0 (0.53, 2.0), *P*=.95 |  |  |  |  |
|  |  | -Race  Black: 0.42 (0.17, 1.1), *P*=.07  Other: 0.29 (0.05, 1.8), *P*=.18 |  |  |  |  |
|  |  | -Education: 1.4 (0.70, 2.8), *P*=.34 |  |  |  |  |
| Need help with any online task | 2.2  (1.3, 3.6)  *P*=.002 | -Age: 1.08 (1.06, 1.1), *P*<.001 | 0.02  (0.01, 0.05)  *P*<.001 | 6 | 195.1  (*P*<.001) | -222.8 |
|  |  | -Gender: 1.1 (0.68, 1.7), *P*=.73 |  |  |  |  |
|  |  | -Race  Black: 2.2 (1.2, 3.9), *P*=.01  Other: 8.7 (2.3, 33.4), *P*=.002 |  |  |  |  |
|  |  | -Education: .22 (.13, .36), *P*<.001 |  |  |  |  |
| Need help to print online materials | 2.7  (1.7, 4.4)  *P*<.001 | -Age: 1.07 (1.05, 1.09), *P*<.001 | 0.01 (0.003, .03)  *P*<.001 | 6 | 179.4  (*P*<.001) | -227.5 |
|  |  | -Gender: 0.87 (0.55, 1.4), *P*=.54 |  |  |  |  |
|  |  | -Race  Black: 3.1 (1.7, 5.9), *P*<.001  Other: 15.9 (4.3, 59.5), *P*<.001 |  |  |  |  |
|  |  | -Education: .24 (.15, .40), *P*<.001 |  |  |  |  |
| Need help to use video | 2.5  (1.4, 4.2)  *P*=.001 | -Age: 1.09 (1.07, 1.1), *P*<.001 | 0.002  (0.0004, 0.008)  *P*<.001 | 6 | 145.1  (*P*<.001) | -184.8 |
|  |  | -Gender: 0.97 (0.58, 1.6), *P*=.91 |  |  |  |  |
|  |  | -Race  Black: 2.3 (1.2, 4.5), *P*=.02  Other: 3.8 (0.94, 15.0), *P*=.06 |  |  |  |  |
|  |  | -Education: 0.50 (.29, .87), *P*=.01 |  |  |  |  |

^a^Dependent variable in multivariate analysis was specific technology access, use, or capability; ^b^Low HL AOR is best fit adjusted odds ratio adjusted for the control variables, low HL is a binary variable in which adequate HL is the reference; ^c^Control variables include age [continuous], gender [binary], race [white, black, other], education [less than any college vs. some college or more]; using Bonferroni correction for the 18 tests, the *P*-value for significance is *P*<.003.
