# Supplementary material for: Evaluating the Need to Address Digital Literacy Among Hospitalized Patients: Cross-Sectional Observational Study
Source: J Med Internet Res. 2020 Jun 4;22(6):e17519. doi: 10.2196/17519 (PMC7303835; doi:10.2196/17519)
Supplement: Multimedia Appendix 2 [file jmir_v22i6e17519_app2.docx]

**Multimedia Appendix 2**. Results of multivariate logistic regression analyses in which low health literacy (HL) adjusted OR was not significant.

| **Dependent variable^a^** | **Low HL AOR^b^ (95%CI)**  ***P*-value** | **Control variables OR^c^ (95%CI),**  ***P*-value** | **Constant**  **(95%CI)**  ***P*-value** | **Df** | **Chi^2^**  **(*P*-value)** | **Log likelihood** |
| --- | --- | --- | --- | --- | --- | --- |
| Own desktop | 0.63  (0.41, 0.98)  *P*=.40 | -Age: 1.0 (0.99, 1.0), *P*=.88 | 0.45  (0.20, 1.0) *P*=.18 | 6 | 33.4 (*P*<.001) | -290.3 |
|  |  | -Gender: 1.1 (0.72, 1.60), *P*=.72 |  |  |  |  |
|  |  | -Race  Black: 0.77 (0.48, 1.24), *P*=.28  Other: 0.52 (0.16, 1.6), *P*=.26 |  |  |  |  |
|  |  | -Education: 2.4 (1.6, 3.7), *P*<.001 |  |  |  |  |
| Own tablet | 0.81  (0.53, 1.2)  *P*=.33 | -Age: .97 (.96, .98), *P*<.001 | 3.5  (1.2, 2.7)  *P*=.002 | 6 | 47.6  (*P*<.001) | -295.4 |
|  |  | -Gender: 1.2 (0.79, 1.7), *P*=.45 |  |  |  |  |
|  |  | -Race  Black: 0.49 (0.30, 0.79), *P*=.003  Other: 0.29 (0.09, 0.94), *P*=.04 |  |  |  |  |
|  |  | -Education: 1.8 (1.2, 2.7), *P*=.004 |  |  |  |  |
| Own smartphone | 0.85  (0.51, 1.4)  *P*=.52 | -Age: .94 (.93, .96), *P*<.001 | 55.7  (18.1, 172)  *P*<.001 | 6 | 75.2  (*P*<.001) | -214.4 |
|  |  | -Gender: 1.2 (0.78, 2.0), *P*=.36 |  |  |  |  |
|  |  | -Race  Black: 0.83 (0.45, 1.5), *P*=.54  Other: 1.4 (0.27, 7.3), *P*=.41 |  |  |  |  |
|  |  | -Education: 1.9 (1.2, 3.1), *P*=.01 |  |  |  |  |
| Wi-Fi at home | 0.67  (0.43, 1.0)  *P*=.07 | -Age: .97 (0.96, 0.98), *P*<.001 | 13.0  (5.0, 33.9)  *P*<.001 | 6 | 58.8  (*P*<.001) | -260.6 |
|  |  | -Gender: 1.1 (0.74, 1.7), *P*=.57 |  |  |  |  |
|  |  | -Race  Black: 0.43 (0.25, 0.78), *P*=.005  Other: 0.55 (0.15, 1.7), *P*=.35 |  |  |  |  |
|  |  | -Education: 2.4 (1.5, 3.7), *P*<.001 |  |  |  |  |
| Unlimited text plan | 0.55  (0.30, 0.99)  *P*=.05 | -Age: 0.93 (0.91, 0.95), *P*<.001 | 170.1  (40.0, 722)  *P*<.001 | 6 | 71.6  (*P*<.001) | -157.8 |
|  |  | -Gender: 1.6 (0.91, 2.8), *P*=.10 |  |  |  |  |
|  |  | -Race  Black: 0.93 (0.46, 1.9), *P*=.85  Other: 1.2 (0.23, 6.9), *P*=.80 |  |  |  |  |
|  |  | -Education: 1.2 (0.66, 2.2), *P*=.56 |  |  |  |  |
| Ever internet use  *Table cont. on next page* | 0.45  (0.24, 0.85)  *P*=.02 | -Age: 0.92 (0.90, 0.94), *P*<.001 | 2790  (430, 18102)  *P*<.001 | 6 | 110.1  (*P*<.001) | -138.4 |
|  |  | -Gender: 0.68 (0.37, 1.3), *P*=.22 |  |  |  |  |
|  |  | -Race  Black: 0.34 (0.14, 0.82), *P*=.02  Other: dropped (16 obs) |  |  |  |  |
|  |  | -Education: 2.3 (1.2, 4.5), *P*=.02 |  |  |  |  |
| Daily internet use | 0.61  (0.39, 0.97)  *P*=.04 | -Age: 0.93 (0.92, 0.95), *P*<.001 | 90.4  (30.0, 272)  *P*<.001 | 6 | 138.9  (*P*<.001) | -246.4 |
|  |  | -Gender: 0.86 (0.56, 1.3), *P*=.50 |  |  |  |  |
|  |  | -Race  Black: 0.49 (0.27, 0.88), *P*=.02  Other: 0.37 (0.10, 1.3), *P*=.12 |  |  |  |  |
|  |  | -Education: 2.5 (1.5, 3.9), P<.001 |  |  |  |  |
| Search health info online | 0.67  (0.42, 1.1)  *P*=.11 | -Age: 0.96 (0.94, 0.97), *P*<.001 | 22.1  (7.6, 64.3)  *P*<.001 | 6 | 61.9  (*P*<.001) | -222.7 |
|  |  | - Gender: 0.97 (0.61, 1.5), *P*=.89 |  |  |  |  |
|  |  | -Race  Black: 0.61 (0.33, 1.1), *P*=.11  Other: 0.42 (0.12, 1.5), *P*=.17 |  |  |  |  |
|  |  | -Education: 2.8 (1.7, 4.6), *P*<.001 |  |  |  |  |
| Post health info online | 0.62  (0.37, 1.0)  *P*=.07 | -Age: 0.97 (0.96, 0.99), *P*<.001 | 1.3  (0.53, 3.3)  *P*=.44 | 6 | 20.4  (*P*=.002) | -226.5 |
|  |  | -Gender: 1.1 (0.7, 1.7), *P*=.66 |  |  |  |  |
|  |  | -Race  Black: 0.97 (0.56, 1.7), *P*=.91  Other: 0.59 (0.15, 2.3), *P*=.45 |  |  |  |  |
|  |  | -Education: 1.2 (0.75, 1.9), *P*=.43 |  |  |  |  |
| Download apps | 0.80  (0.46, 1.4)  *P*=.42 | -Age: .97 (.95, .98), *P*<.001 | 1.8  (0.65, 4.8)  *P*=.27 | 6 | 59.4  (*P*<.001) | -200.7 |
|  |  | -Gender: 1.3 (0.83, 2.2), *P*=.23 |  |  |  |  |
|  |  | -Race  Black: 0.40 (0.23, 0.70), *P*=.001  Other: 0.25 (0.06, 1.0), *P*=.05 |  |  |  |  |
|  |  | -Education: 3.2 (1.9, 5.5), *P*<.001 |  |  |  |  |
| Need help to upload images | 1.4  (0.85, 2.2)  *P*=.20 | -Age: 1.08 (1.06, 1.1), *P*<.001 | 0.009  (.003, .03)  *P*<.001 | 6 | 163.7  (*P*<.001) | -238.9 |
|  |  | -Gender: 1.2 (0.79, 1.9), *P*=.35 |  |  |  |  |
|  |  | -Race  Black: 1.9 (1.1, 3.4), *P*=.03  Other: 5.3 (1.5, 18.9), *P*=.01 |  |  |  |  |
|  |  | -Education: .38 (.24, .62), *P*<.001 |  |  |  |  |
| Need help to open attachment | 1.7  (1.1, 2.8)  *P*=.03 | -Age: 1.08 (1.06, 1.09), *P*<.001 | 0.007  (.002, .02)  *P*<.001 | 6 | 163.7  (*P*<.001) | -229.9 |
|  |  | -Gender: .89 (0.56, 1.4), *P*=.59 |  |  |  |  |
|  |  | -Race  Black: 3.2 (1.7, 6.0), *P*<.001  Other: 4.5 (1.2, 16.3), *P*=.02 |  |  |  |  |
|  |  | -Education: .30 (.19, .49), *P*<.001 |  |  |  |  |
| Need help to use search engine | 2.1  (1.3, 3.5)  *P*=.003 | -Age: 1.07 (1.06, 1.09), *P*<.001 | 0.003  (.0008, .01)  *P*<.001 | 6 | 158.1  (*P*<.001) | -201.1 |
|  |  | -Gender: 1.05 (0.65, 1.7), *P*=.83 |  |  |  |  |
|  |  | -Race  Black: 3.2 (1.6, 6.6), *P*=.001  Other: 3.1 (0.69, 14.5), *P*=.14 |  |  |  |  |
|  |  | -Education: .22 (.13, .39), *P*<.001 |  |  |  |  |

^a^Dependent variable in multivariate analysis was specific technology access, use, or capability; ^b^Low HL AOR is best fit adjusted odds ratio adjusted for the control variables, low HL is a binary variable in which adequate HL is the reference; ^b^Control variables include age [continuous], gender [binary], race [white, black, other], education [less than any college vs. some college or more]; using Bonferroni correction for the 18 tests, the *P*-value for significance is *P*<.003.
